# Advances in the Cellular and Molecular Mechanisms of Cardiovascular Diseases

**DOI:** 10.3390/cells15171587

**Published:** 2026-09-01

**Authors:** Katie S. Wraith, Timothy M. Palmer

**Affiliations:** Centre for Biomedicine, Hull York Medical School, University of Hull, Hull HU6 7RX, UK; katie.wraith@hyms.ac.uk

## Introduction

Cardiovascular diseases (CVDs) remain the leading cause of death worldwide despite major advances in prevention, diagnosis and treatment. While improvements in risk factor management and acute clinical care have reduced mortality in some patient groups, the global burden of atherosclerosis, heart failure, vascular disease and age-associated cardiovascular dysfunction continues to rise. Addressing this challenge requires a deeper understanding of the cellular and molecular mechanisms that drive disease initiation, progression and tissue remodelling. This Special Issue of *Cells*, “Advances in the Cellular and Molecular Mechanisms of Cardiovascular Diseases”, brings together a collection of research studies and narrative reviews spanning cardiovascular metabolism, inflammation, cellular signalling, vascular remodelling and gene regulation, highlighting emerging therapeutic opportunities spanning multiple cardiovascular pathologies.

A recurring theme throughout this Special Issue is the central role of metabolic regulation in determining cardiovascular cell phenotype and function. Growing evidence indicates that alterations in mitochondrial activity and substrate utilisation are not simply consequences of disease but active drivers of pathological remodelling [1,2]. Chempon and colleagues (Contribution 1) [3] demonstrate that metformin suppresses cardiac fibroblast differentiation and cellular senescence through preservation of fatty acid β-oxidation and mitochondrial bioenergetics, providing new mechanistic insight into the anti-fibrotic actions of this widely used therapeutic agent. Similarly, Bolanle et al. (Contribution 2) [4] identify JAK- and MEK-dependent regulation of mitochondrial activity in diabetic saphenous vein smooth muscle cells, highlighting signalling pathways that may contribute to vein graft failure and vascular dysfunction in diabetes. Together, these studies reinforce the concept that metabolic pathways represent attractive targets for therapeutic intervention in cardiovascular disease.

The collection also highlights the importance of inflammatory signalling networks in cardiovascular pathology. Chronic vascular inflammation is a major driver of remodelling, aneurysm formation and tissue injury, yet the mechanisms linking inflammatory stimuli to cellular dysfunction remain incompletely understood [5]. Spartano et al. (Contribution 6) [6] demonstrate that pharmacological inhibition of CXCR2/CXCL1 signalling with ladarixin attenuates several pathological responses in endothelial and vascular smooth muscle cells exposed to inflammatory stimuli relevant to abdominal aortic aneurysm. These findings support a growing body of evidence implicating chemokine signalling pathways as potential therapeutic targets for vascular disease. In parallel, Garg et al. (Contribution 7) [7] identify prostaglandin D2 synthase as a previously unrecognised mediator of aldosterone–mineralocorticoid receptor signalling in the heart, establishing a novel link between hormonal signalling, cardiac hypertrophy and fibrotic remodelling.

Another important theme emerging from this Special Issue is the increasing recognition of the fundamental roles post-transcriptional mechanisms play in cardiovascular adaptation to stress and injury. Itani and co-workers (Contribution 5) [8] demonstrate that the RNA-binding proteins Roquin-1 and Roquin-2 regulate cardiac post-infarction remodelling through control of microRNA stability and downstream transcriptional networks. These findings add to a rapidly expanding literature demonstrating that RNA-dependent regulatory pathways are major determinants of cardiovascular cell fate and function and may provide opportunities for highly specific therapeutic targeting [9]. Narrative reviews evaluating the roles of the Notch pathway in atherosclerosis (Contribution 9) [10], new findings on how inflammation drives acute coronary syndromes (Contribution 10) [11] and the molecular impact of catheter ablation on atrial fibrillation (Contribution 11) [12] elegantly summarise current research in these areas.

Taken together, the studies published in this Special Issue show that cardiovascular diseases arise through the complex interaction of metabolic dysfunction, inflammatory signalling, altered cellular plasticity and dysregulated gene expression. Importantly, these processes are interconnected and frequently converge on common pathological endpoints, including fibrosis, vascular remodelling, cellular senescence and impaired tissue repair. The work presented here therefore advances our understanding of cardiovascular disease across multiple biological scales, from intracellular signalling pathways to whole-organ remodelling.

Collectively, these articles also provide compelling evidence that mechanistic cardiovascular research will continue to generate clinically relevant insights with significant translational potential. By identifying new molecular targets, clarifying disease pathways and revealing previously unrecognised regulatory mechanisms, the contributions in this Special Issue expand the foundation upon which future diagnostic and therapeutic strategies will be built. As the burden of cardiovascular disease continues to grow globally, such interdisciplinary and mechanistically driven research will be essential for translating mechanistic insights in the development of more effective approaches to prevention, diagnosis and treatment.
